# What's a SNP between friends: The influence of single nucleotide polymorphisms on virulence and phenotypes of *Clostridium difficile* strain 630 and derivatives

**DOI:** 10.1080/21505594.2016.1237333

**Published:** 2016-09-21

**Authors:** Mark M. Collery, Sarah A. Kuehne, Shonna M. McBride, Michelle L. Kelly, Marc Monot, Alan Cockayne, Bruno Dupuy, Nigel P. Minton

**Affiliations:** a Clostridia Research Group, BBSRC/EPSRC Synthetic Biology Research Center (SBRC), School of Life Sciences, University of Nottingham, Nottingham, UK; b NIHR Biomedical Research Unit in Gastrointestinal and Liver Diseases at Nottingham University Hospitals NHS Trust, University of Nottingham, Nottingham, UK; c Department of Microbiology and Immunology, Emory Antibiotic Resistance Center, Emory University, Atlanta, GA, USA; d Laboratoire Pathogenèse des Bactéries Anaérobies, Institut Pasteur, Paris, France

**Keywords:** ClosTron, motility, mutation, single nucleotide polymorphism, sporulation, toxin expression, virulence

## Abstract

*Clostridium difficile* is a major cause of antibiotic induced diarrhea worldwide, responsible for significant annual mortalities and represents a considerable economic burden on healthcare systems. The two main *C. difficile* virulence factors are toxins A and B. Isogenic toxin B mutants of 2 independently isolated erythromycin-sensitive derivatives (630E and 630Δ*erm*) of strain 630 were previously shown to exhibit substantively different phenotypes. Compared to 630, strain 630E and its progeny grow slower, achieve lower final cell densities, exhibit a reduced capacity for spore-formation, produce lower levels of toxin and are less virulent in the hamster infection model. By the same measures, strain 630Δ*erm* and its derivatives more closely mirror the behavior of 630. Genome sequencing revealed that 630Δ*erm* had acquired 7 unique Single Nucleotide Polymorphisms (SNPs) compared to 630 and 630E, while 630E had 9 SNPs and a DNA inversion not found in the other 2 strains. The relatively large number of mutations meant that the identification of those responsible for the altered properties of 630E was not possible, despite the restoration of 3 mutations to wildtype by allelic exchange and comparative RNAseq analysis of all 3 strains. The latter analysis revealed large differences in gene expression between the 3 strains, explaining in part why no single SNP could restore the phenotypic differences. Our findings suggest that strain 630Δ*erm* should be favored over 630E as a surrogate for 630 in genetic-based studies. They also underline the importance of effective strain curation and the need to genome re-sequence master seed banks wherever possible.

## Introduction

*Clostridium difficile* is a Gram-positive, anaerobic spore-forming bacterium capable of causing a range of diseases from mild diarrhea to potentially fatal toxic pseudomembranous colitis. The toxigenic effects of *C. difficile* are caused by the activities of 2 large, glucosylating toxins. The two toxins are 308kDa (toxin A) and 270kDa (toxin B) in size^1-3^ and are encoded by the chromosomally located genes *tcdA* and *tcdB*, respectively. Both are cytopathic to cultured cells due to disruption of the cytoskeleton, although TcdB is thought to be up to 1000-times more potent.^1^ Historically, toxin A was regarded as the main causative agent of the symptoms of *C. difficile* infection (CDI). Pivotal data was provided by Lyerly et al.^4^ who were only able to detect disease when hamsters were subject to intragastric challenge with purified TcdA alone and not with TcdB. The latter could, however, cause disease symptoms if prior damage to the mucosa had been inflicted by co-administration of sub-lethal concentrations of toxin A. Furthermore, co-administration of both toxins led to more severe disease symptoms. To accommodate these data, it was generally accepted that both toxins acted in concert to bring about disease symptoms, with toxin A leading to the initial damage to the colon allowing the subsequent access of the more potent toxin B.

During the 1990s *C. difficile* strains were isolated from symptomatic patients that only produced toxin B (A-B+).^5,6^ These findings suggested that toxin B, at least in certain strains, is capable of causing disease without the help of toxin A. It has been reported since that toxin B, in A-B+ strains, is modified and seems to be an evolutionary hybrid of *C. difficile* toxin B and *Clostridium sordellii* lethal toxin.^7^

With the development of genetic systems, assumptions of the relative importance of the 2 toxins could be tested through the creation, and *in vivo* assay, of isogenic mutants in which production of either toxin had been ablated. Initial findings made by Lyras et al.^*8*^ appeared to turn the perceived view on its head, through the demonstration that a *tcdA* mutant producing TcdB alone (A-B+) was capable of causing disease in the hamster model while a *tcdB* mutant producing only TcdA (A+B-) did not. These data were, however, almost immediately questioned by a second study conducted in the Minton laboratory^9^ showing that both *tcdA* and *tcdB C. difficile* mutants, and therefore TcdA and TcdB alone, were independently capable of causing disease. Interestingly, a strain has recently^10^ been isolated from a clinical case of CDI, that only produces TcdA (A+B-).

The possible reasons for the observed difference in outcomes of the 2 studies have been discussed previously.^11^ Both studies agree on the virulence potential of toxin B, but uncertainties remain about the different outcomes concerning the effects of toxin A. In the work presented here, we have hence focused on comparisons of the parental strains and the strains only producing toxin A (A+B-). In essence, both sets of mutants were generated by insertional inactivation of the toxin genes of the *C. difficile* strain 630^12^ and, once created, were tested in the hamster infection model. However, in order to implement the available gene tools in strain 630 (at the time the only strain for which a genome sequence was available), it was necessary to first isolate a variant that had become sensitive to erythromycin, thereby allowing the use of an *ermB* gene as a selective, genetic marker. Both studies used such an erythromycin-sensitive derivative of strain 630, but they were independently isolated. In our study (Minton group),^9^ we used the strain 630Δ*erm*, isolated in the Mullany laboratory (UCL, London, UK) after 30 repeated subcultures of strain 630 in non-selective media.^13^ In parallel, the Rood laboratory (Monash, Australia) independently isolated the erythromycin sensitive strain JIR8094 (also referred to as 630E),^14^ through an undisclosed number of subcultures of strain 630 in non-selective media. Both strains are reported to possess the same specific deletion of *ermB*.^13,14^

We have previously hypothesized^11^ that the different outcomes of the 2 studies^8,9^ are a direct consequence of the use of the 2, independently isolated erythromycin-sensitive strains, 630Δ*erm* and 630E. We suggested that during repeated subculture, ancillary mutations arose which impacted on the virulence potential of one or other of the 2 strains in the presence of different toxin gene alleles. In the current piece of work, we have set out to test this hypothesis. We have undertaken side-by-side comparisons of 630Δ*erm* and 630E, and the A+B- mutant derivatives, in a variety of assays to establish phenotypic differences. In parallel, we have determined the genome sequences of the various strains used in the 2 studies.^8,9^ Then, we have used our newly developed allelic exchange methodologies^15^ to correct a number of SNPs in strain 630E back to wild-type and assessed the consequences. Furthermore we have performed RNAseq experiments comparing the transcriptome of 630, 630Δ*erm* and 630E at 3 different time points. The RNA data were related to the whole genome data to draw our final conclusions.

## Results

### Generation of a ClosTron insertion in *tcdB* of 630E

Although considered unlikely, the possibility existed that mutants made by the insertion of a plasmid element carrying *ermB*^14^ might behave differently to an equivalent mutant made by the insertion of a group II intron incorporating *ermB*.^16^ Our initial step was, therefore, to create a *tcdB* mutant of strain 630E using ClosTron technology. Accordingly, the ClosTron plasmid pMTL007C-E2::Cdi-tcdB-1511a that had previously been used to generate strain 630Δ*erm* A+B-^9^ was used to create an equivalent mutant in strain 630E as described.^9^ The resulting mutant, 630E A+B-CT, was verified by PCR, Sanger sequencing and shown by Southern blot to carry a single group II intron insertion (Fig. S1A). Parental strains, original A+B- mutants and the newly obtained mutant were tested for production of toxin A in a Western blot (Fig. S1B). As expected all strains produced toxin A.

### Phenotypic characterization of strains

**Figure 1.**
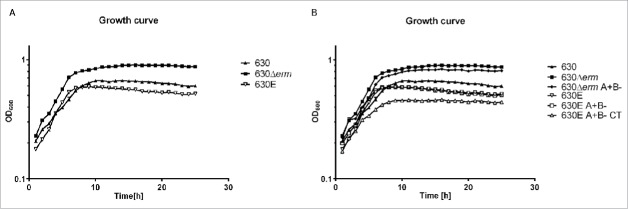
Growth curves of strain 630 and its derivatives. A. 630 (630), 630Δ*erm* and 630E were grown in TY-broth for 24 h in a 96 well plate reader. The optical density at 600 nm was measured every 30 min. B. This graph shows the same growth as A. and in addition the growth of derivatives 630Δ*erm* A+B-, 630E A+B- and 630E A+B- CT.

**Figure 2.**
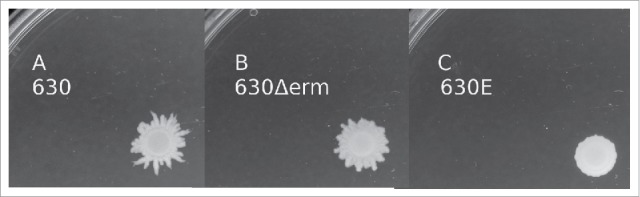
Motility assays. The assay was carried out by inoculating overnight cultures onto motility agar plates and incubating anaerobically for 48 h. Strains 630, 630Δ*erm* and 630E were compared for their ability to swarm.

In order to establish whether all strains were phenotypically identical a range of assays were performed, comparing growth, motility and spore properties. An analysis of growth rates using the procedure described in Materials and Methods showed that strain 630Δ*erm* and derivatives grew to the highest optical density, closely followed by strain 630 and 630E, and derivatives thereof (Fig. 1A and 1B). The data clearly demonstrated that strain 630Δ*erm* and its 630Δ*erm* A+B- derivative had relatively higher growth rates and achieved higher optical densities (p < 0.0001, unpaired t-test at 24 h) than strain 630E and its derivatives, with strain 630E A+B-CT growing the least (Fig. 1B). It was also apparent, shown by plate motility assay (Fig. 2), that strains 630 and 630Δ*erm* were motile, while 630E was not. Only 630 and 630Δ*erm*, but not 630E, form pseudopod-like structures, which are characteristic for swarming motility in bacteria.

**Figure 3.**
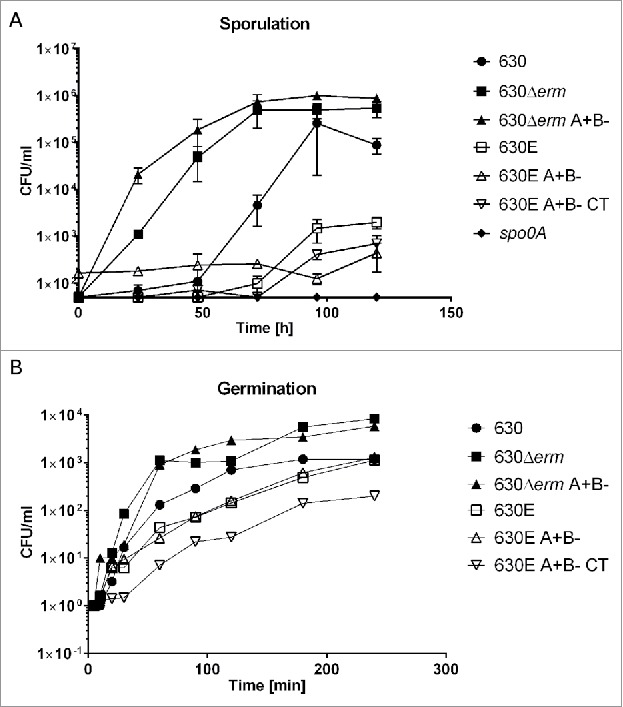
Sporulation and Germination. A. Sporulation over 120 h comparing heat treated CFUs of strains 630, 630Δ*erm*, 630Δ*erm* A+B-, 630E, 630E A+B- and 630E A+B- CT with a non-sporulating control (*spo0A*). B. The extent of germination of the indicated strains was measured over 250 min as the ability of germinated spores to form colonies on plates lacking taurocholate.

Following the protocols of Burns et al,^17^ comparative differences in the numbers of colony forming units (CFUs) obtained following heat shock were assessed between the strains, as a crude estimate of spore formation.^18^ On this basis, strains 630Δ*erm* and 630Δ*erm* A+B- produced a greater numbers of spores than 630E and its derivatives, which failed to produce any spores until 72 h. The total number of CFU/mL at this time point was 10^3^ times fewer than that obtained with 630Δ*erm* or 630Δ*erm* A+B- (Fig. 3A). Interestingly parental strain 630 produced very few spores before 72 h, but spore counts increased from 72 h onwards and reached similar levels to strains 630Δ*erm* and 630Δ*erm* A+B- by the end of the experiment. The reduction in spore formation may in part be due to the observed reduction in OD as the 630E strains enter stationary phase, which might also explain their predilection to flocculate. Indeed comparing percentage sporulation (relative to vegetative cell count), confirmed the observation that 630Δ*erm* and 630Δ*erm* A+B- have a higher sporulation frequency than both 630E (and derivatives) and strain 630.

The germination of the 630E strains was also comparatively reduced and did not reach the same level as that of 630Δ*erm* and its progeny. At the last time point (240 min) strains 630E and 630E A+B- reach the same level of CFU/ml as strain 630. The observed delay could be due to the previously observed reduced cell growth of the 630E strains (Fig. 3B).

### Toxin production

**Figure 4.**
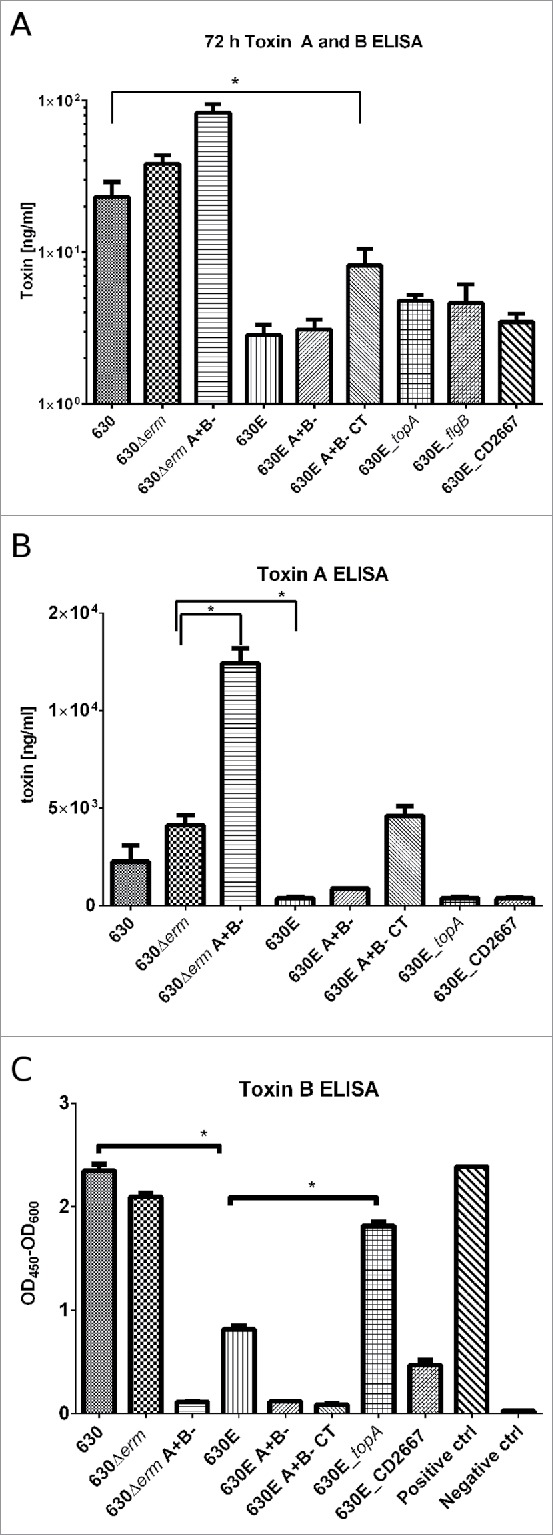
Toxin ELISAs. A. The *C. DIFFICILE TOX A/B II*™ ELISA assay kit from TechLab was used to measure combined toxin A and B in strains 630, 630Δ*erm*, 630Δ*erm* A+B-, 630E, 630E A+B-, 630E A+B- CT, 630E_*topA*, 630E_*flgB* and 630E_CD2667 grown in TY for 72 h. B. and C. Toxin ELISAs TGCbiomics, measuring the toxins separately were used to quantify toxin A (B) and toxin B (C) produced by strains 630, 630Δ*erm*, 630Δ*erm* A+B-, 630E, 630E A+B- and 630E A+B- CT grown in TY for 72 h. Statistics were performed using one-way ANOVA with Fisher's LSD test.

Measurements of the amounts of toxin being produced by 630E and 630Δ*erm* and their derivatives were undertaken using both the *C. DIFFICILE TOX A/B II*™ ELISA assay kit from TechLab, measuring toxin A and B, and kits from TGCbiomics, specifically measuring either only toxin A or only toxin B. The results of the 72 h time point are shown in Fig. 4.

As shown in Fig. 4A, toxin production of 630, 630Δ*erm* or 630Δ*erm* A+B- clustered together, as did toxin production of 630E, 630E A+B- or 630E A+B- CT with the latter 3 showing no statistical differences between them (P > 0.05, one-way ANOVA with Fisher's LSD test). There was, however, a statistically significant difference between the first 3 strains (630, 630Δ*erm* or 630Δ*erm* A+B-) and the second set of 3 strains (630E, 630E A+B- or 630E A+B- CT) (P < 0.0001). The *C. DIFFICILE TOX A/B II*™ ELISA does not differentiate between toxin A and B. In order to be able to quantify each toxin, the kits from TGCbiomics were used (Fig. 4B and 4C). The toxin A ELISA showed significantly higher production in strain 630Δ*erm* compared to 630E (p < 0.0016) and also confirmed the previous observations that strains with impaired *tcdB*, produce more toxin A^8,9^ (Fig. 4B). No toxin B production was seen, as expected, in the *tcdB*-mutants. Strains 630 and 630Δ*erm*, however, both produced significantly more toxin B than 630E (p < 0.0001) (Fig. 4C).

### Whole genome sequencing

To establish whether strains 630E and 630Δ*erm*, and derivatives, contained any additional changes to the *ermB* gene deletion, relative to the parent strain 630, the following strains were sequenced using Next Generation Sequencing platforms: 630E A+B-, 630E A+B- CT on Illumina HiSeq (GATC, Germany) and 630Δ*erm* A-B on a Roche 454 (Deepseq, University of Nottingham, UK) and the data compared to the published genome of the parental strain 630^12^ and previously sequenced 630Δ*erm*Δ*pyrE*.^19^ We used a frequency of 70% as a cut-off for SNP calling and found multiple SNPs, InDels and other minor changes, both common and unique to 630, 630E and 630Δ*erm*. In total, 2 SNPs in coding regions with non-synonymous changes were found that were common to all 3 strains (in CD630_11900, encoding an acyl-CoA N-acyltransferase where SNP changes phenylalanine133 to leucine and in CD630_13880, a pseudo gene where a frameshift is introduced). In addition to these, we found in both 630 and 630Δ*erm* strains 3 SNPs (2 in intergenic regions and one in a coding region of CD630_2667, encoding the BC domain of a glucose PTS, changing valine228 to isoleucine). 630Δ*erm* had 7 unique changes compared to 630 and 630E (including 6 non-synonymous SNPs in coding regions), while 630E had 11 SNPs (with 9 non-synonymous SNPs in coding regions) not found in the other 2 strains. SNPs were confirmed by Sanger sequencing and thereafter by RNAseq data (see below). Indeed, the SNPs found in the DNA-seq data were validated by using the RNA-seq sequence reads mapped on the genome sequence with Bowtie2^20^ with each position being checked using Tablet.^21^ A complete list of SNPs and other small changes are indicated in Table 1 and in Table S1. During the preparation of this manuscript a new sequence of 630 was published by Riedel *et al*.^22^ We incorporated their data into Table 1 (and Table S1). Overall this new sequence shows very few disparities to the original one. However two SNPs found in our data were attributed to mistakes in the original sequence (in CD630_17670 and CD630_31561). Another paper was recently published by van Eijk *et al*.,^23^ resequencing 630Δ*erm*. Overall there are very few discrepancies between their data and our findings, confirming the quality of both data sets. We have incorporated their findings into Table S1.

**Table 1.** Single Nucleotide Polymorphisms (SNPs) and other changes found after re-sequencing.

Gene	Description	Position	630 ^12^	630 ^22^	630	C	F	630Δ*erm*	C	F	630E	C	F	AA
630E														
CD630_07610	Putative ATP-dependent RNA helicase	933139	G	—	—			—			T	199	100	Asp136Tyr
CD630_14040	Putative oligopeptide transporter	1626977	A	—	—			—			G	187	100	Glu536Gly
CD630_20270	N-carbamoyl-L-amino acid hydrolase	2339506	G	—	—			—			A	73	100	Gly373Glu
CD630_26670	PTSG-BC	3079815	A	—	—			—			C	165	100	*524Glu
CD630_26270	Hypothetical protein	3034953	C	A	A			A			—	156	100	Gly68Cys
CD630_33790	conjugative transposon protein	3951559	C	—	—			—			A	307	100	Glu63Asp
CD630_12740	*topA*	1480649	C	—	—			—			T	146	100	Gln386*
CD630_29430	Putative phage replication protein	3422569	T	—	—			—			C	195	100	Asn210Asp
IG	Intergenic region	309208	—	—	—			—			INV	129	41	
IG	Intergenic region	3528736	G	—	—			—			T	94	100	
630Δ*erm*														
CD630_19070	*eutG*	2209236	G	—	—			A	127	97				Gly252Glu
CD630_35650	GntR family transcriptional regulator	4166495	G	—	—			A	182	100	—			Ala91Val
IG	Intergenic region	2937176	C	—	—			A	173	100	—			
IG	Intergenic region	3005866	T	—	—			G	156	100	—			
IG	Intergenic region	3591103	G	—	—			A	211	100	—			
CD630_12140	*spo0A*	1413057	—	—	—			AGAATGT-AGGAAA-TATAG	112	40	—			Insertion
CD630_08260	Ferric uptake regulator	1000995	A	—	—	100	100	G	153	100	—	170	98	Thr41Ala
630														
CD630_32450	*prdR*	3797112	C	—	T	117	100	—			—			Glu261Lys
CD630_02050	Transcription antiterminator, PTS operon regulator	268934	G	—	T	123	97	—			—			Gly165Cys
630 and 630Δ*erm*														
CD630_2667	PTSG-BC	3080703	C	—	T	75	100	T	141	100	—			Val228Ile
IG	Intergenic region	2203033	A	—	T	105	100	T	174	99				
IG	Intergenic region	4007463			C	10	100	C	62	100				
630 and 630Δ*erm* and 630E														
CD630_11900	acyl-CoA N-acyltransferase	1391850	T	—	C	118	100	C	133	100	C	143	100	Phe133Leu
CD630_13880	pseudo	1607453	INS ^1^	—	T	119	94	T	175	94	T	124	88	Thr16fs
Mistake in original sequence														
CD630_17670	*gapB*	2044514	C	G	G			G			G			
CD630_31561	pseudo	3686535	INS ^1^	A	A			A			A			

The table shows the Single Nucleotide Polymorphism (SNP) changes in 630Δ*erm* and 630E compared to the reference 630^12^ and also the new annotation by Riedel *et al* (The column ‘Gene’ represents the gene (or intergenic region (IG)) in which the change occurs, the column ‘position’ indicates the exact nucleotide position of the change.).^22^ It also contains SNP frequency (F) and genomic coverage (C) as well as the resultant amino acid change (AA). No change from the original 630 annotation^12^ is represented by a dash (–).

It may be assumed, that during the repeated subculture of strain 630 undertaken in the Mullany^13^ and Rood^14^ laboratories, sub-populations within the culture were isolated carrying SNPs. However, it seems improbable that the 2 SNPs (Table 1), common to all 3 strains, arose independently. Rather we hypothesize that these SNPs might be sequencing mistakes. This theory gains weight through the new sequencing data by Riedel *et al*.^22^ Two SNP changes which we identified originally between the published 630 sequence and our data were confirmed by Riedel et al to also be the sequence of their 630 seed stock. Unfortunately the genome announcement^22^ does not state the exact source of their 630 strain. As mentioned above another 3 SNPs were only found in 630 and 630Δ*erm*, 2 of these are in intergenic regions which showed no expression in our RNAseq experiment, and the third is located in a PTS gene in 630 and 630Δ*erm* (position 3080703, Val_228_Ile). Rather than having occurred independently it is more likely that these SNPs arose in the Mullany laboratory, subsequent to provision of chromosomal DNA to the Sanger Center for determination of the 630 genome sequence,^12^ and before the strain 630 was passaged to obtain 630Δ*erm*. At the time, *C. difficile* strains in the Mullany laboratory were routinely stored at 4°C as Robertson's Cooked Meat stocks, as opposed to being frozen at −80°C in 10% glycerol (A.R. Roberts, personal communication). On this basis, the traditional microbiological practice of using Robertson's Cooked Meat to curate strains might not be ideal as strains are not entirely dormant and genome changes can occur over time. The SNPs that were found to be unique to 630Δ*erm* and 630E (n = 8 and n = 11, respectively) can be assumed to have been accrued at some point after the 2 630 populations diverged, that is when the strain was sent to the Rood laboratory. It is most likely, although not certain, that the majority, if not all of the strain-specific SNPs arose during the repeated subculture experiments undertaken to isolate the *ermB* deletion strains 630E and 630Δ*erm*.

Changes specific to 630Δ*erm* include SNPs in 3 intergenic regions, which all have been determined with a coverage of over 150 and 100 % frequency (see Table 1). The other 5 changes comprise 4 non-synonymous SNPs and an insertion. The insertion has previously been reported by Rosenbusch *et al*.^24^ and was confirmed by van Eijk *et al*.^23^ and is an 18 bp duplication in *spo0A*, the master regulator of sporulation. This insertion might be responsible for the reduced sporulation frequency seen in strain 630 and also in 630E and derivatives, which do not carry this duplication (Fig. 3A). The SNPs have been found in the following genes: CD630_08260, encoding a ferric uptake regulator (*perR* homolog) (Thr_41_Ala); CD630_19070, encoding an alcohol dehydrogenase homolog (*eutG*) (Gly_252_Glu); and CD630_35630, encoding a transcriptional regulator of the GntR family (Ala_91_Val).

In contrast, strain 630E contains a larger number of non-synonymous SNPs including changes that result in nonsense mutations and in one case the inversion of a small segment of DNA preceding a flagella operon. We found 2 changes in intergenic regions, one with 100 % frequency and a coverage of 94 (position 3528736); the other at a much lower frequency (41 %), but confirmed a 150 bp inversion by Sanger sequencing in the promoter region of *flgB*, the first gene in a F3 flagella operon (early flagella genes). Non-synonymous SNPs were found in CD630_07610, encoding a putative RNA helicase (Asp_136_Tyr); CD630_14040, encoding an oligopeptide transporter (Glu_536_Gly); CD630_20270, encoding a hydrolase (Gly_373_Glu); CD630_29430, encoding a phage replication protein (Asn_210_Asp); and CD630_33790, encoding a conjugative transposon protein (Glu_63_Asp). Finally, there is another SNP at position 3034953, in gene CD630_26270 (Gly_68_Cys), encoding a conserved hypothetical protein. Interestingly the new genome sequence from Eijk et al.^23^ suggested an “A” at position 3034953 in contrast to the earlier annotation suggesting “C.” The new annotation is in line with our RNAseq data (Table S1) and taken into account our sequencing data (Table 1) suggests that this is indeed a mutation in 630E and was miss-annotated in the original sequence. In one instance the nucleotide substitution resulted in the creation of a nonsense, stop codon, and as a consequence a severe, premature truncation of the encoding protein. Thus, the stop codon introduced into CD630_12740 encoding a topoisomerase I (*topA*) homolog (Gln_386_*) truncated the protein from 695 amino acids to 385 amino acids. Conversely, in the case of the glucose PTS operon *ptsG*-BC, the conversion of the stop codon of *ptsG-B* gene (CD630_26670) to a Glu codon (*_524_Glu) resulted in its fusion to the coding region of the immediately downstream *ptsG-C* gene.

### Virulence testing of 630Δ*erm*, 630E and mutants using an *in vivo* model

In order to confirm previous data and to rule out differences in experimental set up in different laboratories, the virulence of 630E and derivatives was assessed using the hamster infection model in our laboratory (University of Nottingham) as previously described.^9^

**Figure 5.**
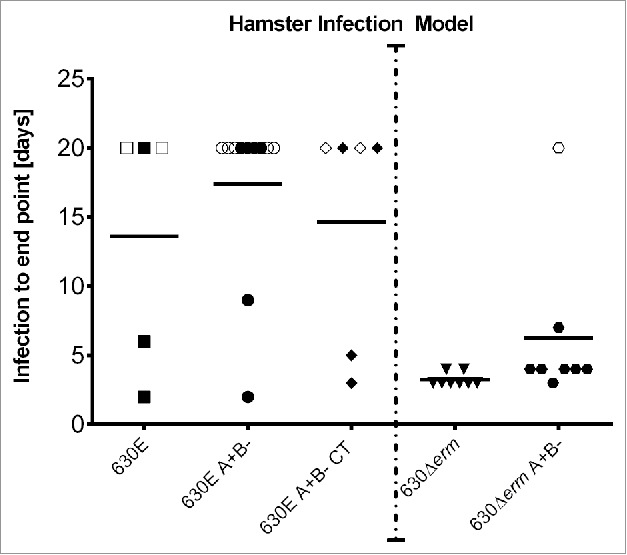
Infection to endpoint in the Hamster infection model. Groups of Golden Syrian Hamsters were challenged with *C. difficile* 630E (5 hamsters), 630E A+B- (11 hamsters) and 630E A+B- CT (6 hamsters). The graph represents the time from inoculation to endpoint. The maximal duration of the experiment was set to 20 d. Animals represented in open symbols, have not been colonized despite challenge or lost colonization before day 20. Details can be seen in Table 1. The dotted line separates this experiment from data obtained by Kuehne et al.,^9^ which are represented here as a comparator. In that study 8 hamsters were infected with *C. difficile* 630Δ*erm* and another 8 hamsters with 630Δ*erm* A+B-.

Figure 5 shows the times from infection to endpoint (in days) for the hamsters infected with 630E, 630E A+B- and 630E A+B- CT. For comparative purposes data for infection with 630Δ*erm* and 630Δ*erm* A+B- from a previous study^9^ is also included. The latter emphasizes the fact that all 8 hamsters infected with strain 630Δ*erm* were colonised and succumbed to *C. difficile* disease (with an average time of 3.25 d from infection to endpoint). This is in direct contrast to what is observed with 630E where of the 5 animals successfully infected, only 3 were colonised till the respective endpoints and of these, 2 succumbed to disease (at day 2 and 6). Two animals lost colonisation after days 15 and 18, respectively.

In our previous study^9^ 7 of the 8 animals infected with 630Δ*erm* A+B- (as also shown in Fig. 5), succumbed to disease with an average time to death of colonised hamsters being just under 2 d. One animal showed no signs of disease until the experimental endpoint, but was found not to have been colonised. Here of the 11 animals infected with the equivalent mutant of strain 630E (630E A+B-), only 2 animals succumbed to CDI (on day 2 and 9). Four animals in this group were never colonised, one lost colonisation after day 3 and the others were colonised till endpoint. Six animals were infected with 630E A+B- CT, and of these 2 hamsters developed infection (day 3 and 5). Two of the surviving animals lost colonisation after day 15 and 18 respectively. (Fig. 5 and Table S2).

The difference between the average time to death of all hamsters administered 630Δ*erm* and 630Δ*erm* A+B- was found not to be statistically significant (one-way ANOVA, p = 0.5355) (results from Kuehne *et al.*^9^). Similarly, the differences between the average times to death of all animals administered 630E, 630E A+B- and 630E A+B- CT was not statistically significant (one-way ANOVA, p = 0.8919). In contrast, the difference between the 630Δ*erm* strain (and derivative) and the 630E strain (and derivatives) was statistically significant (one-way ANOVA, p > 0.0001).

### Correction of SNPs in strain 630E

In view of the large number of SNPs present in strain 630E, it was impractical to change them all back to the 630 parental sequence. We therefore selected just 3 specific mutations present in 630E and converted them back to the sequence present in the parental strain, 630.

Our principal target was to remove the stop codon from within the topoisomerase I gene, CD630_12740, as this enzyme plays a central role in the regulation of DNA negative supercoiling and its inactivation is likely to result in extensive pleiotropic effects. Indeed, in some bacteria its inactivation is lethal.^25-27^ Moreover, bacterial genes related to pathogenesis and virulence have been shown to be sensitive to *topA* mutation in *E. coli*,^28^ *S. flexneri,*^29^ *Yersinia enterocolitica*^30^ and *Salmonella*.^31^ We therefore converted the “T” nucleotide at position 1480649 in 630E back to an “A” nucleotide, thereby removing the nonsense stop codon and allowing the production of full length native topoisomerase enzyme.

As a second target we elected to correct the inversion of DNA upstream of the F3 flagella operon. As strain 630E is non-motile, and as the inverted region encompasses the non-coding region immediately upstream of the *flgB* gene, it is likely to have disrupted the promoter responsible for both *flgB* expression and the genes in the downstream operon. The inversion is therefore likely to be the principal cause of the loss of motility in 630E. Furthermore, factors affecting flagella expression can also influence toxin expression levels.^32,33^

Finally, we sought to correct the fusion of the 2 PTS components *ptsG-B* and *ptsG-C*, by resurrection of the stop codon of *ptsG-B* through the conversion of the “C” nucleotide at position 3079815 back to an “A” nucleotide. As glucose is known to affect toxin production, through catabolite repression,^34,35^ it was reasoned that this particular SNP could be affecting toxin expression, and therefore virulence.

The plasmids carrying the 630 wildtype alleles necessary for the correction of the 3 targeted SNPs were assembled as described in Materials and Methods and then used to effect the replacement of the 630E mutant alleles by allelic exchange.^15^ To verify that the mutant clones obtained were correct, each targeted region was amplified by PCR using appropriate oligonucleotide primers and the DNA fragments obtained subjected to Sanger sequencing on both DNA strands. In every case, clones carrying the desired “corrected” sequence were obtained. The new strains were named after the genes or regions that were corrected, namely 630E_*topA*, 630E_CD2667 and 630E_*flgB*, respectively.

To assess the effects of the changes on the characteristics of the mutant strains, growth rate, sporulation and germination, motility, and *in vitro* cytotoxicity and toxin production (ELISA) were measured. None of the 3 corrected mutants exhibited any difference in growth rate compared to the parental strain 630E (data not shown). Similarly, sporulation and germination remained unaffected (data not shown). Toxicity testing revealed no difference to 630E using the *C. DIFFICILE TOX A/B II*™ ELISA assay kit from TechLab (Fig. 4A). To quantify toxin A and toxin B individually the ELISA kits from TGCbiomics were used to assay 630E_*topA* and 630E_CD2667 (Fig. 4B). No differences were measured for toxin A, but the strain 630E_*topA* showed significantly higher levels of toxin B than the parental strain 630E.

### Transcriptomic comparison of 630, 630Δ*erm* and 630E

**Figure 6.**
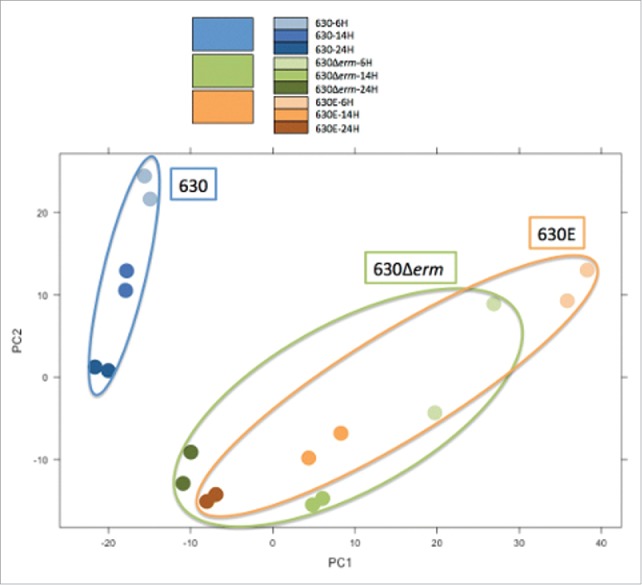
Principal Component Analysis (PCA). The Principal Component Analysis (PCA) visualizes the variance of the data in a single graph. The axis represent the 2 largest variances of the data; PC1 accounts for 42% and PC2 accounts for 21%, that means that 63% of the total variance of the dataset is explained in this graph. The third component accounts for less than 10% and further components have a value that falls rapidly. The PCA represents the RNAseq data (at 3 different time points, 6, 14 and 24 h) in duplicate for 630 (blue), 630Δ*erm* (green) and 630E (orange). The different time points are represented as dots in the different shades of the respective color as indicated in the color legend.

**Figure 7.**
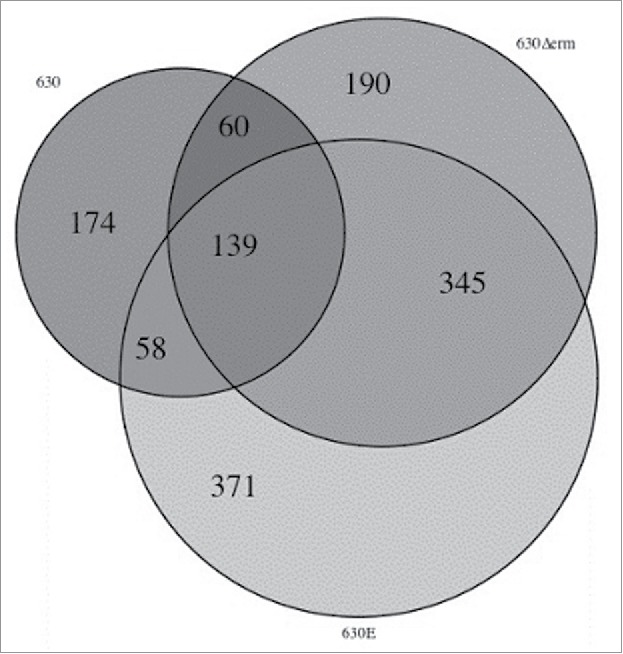
Venn Diagram representing differentially expressed genes in the 3 different strains. The diagram summarizes the output from the RNAseq data, comparing strains 630, 630Δ*erm* and 630E. It depicts all differentially expressed genes and shows how many genes are differentially expressed in all strains, in 2 of the strains or are unique to just one strain.

RNA was extracted from strains 630, 630Δ*erm* and 630E at 6, 14 and 24 h and used in an RNAseq experiment as described in Materials and Methods. The Principal Component Analysis (PCA) (Fig. 6) showed that strain 630Δ*erm* and 630E are closely correlated on a transcriptional level which is significantly separated from 630. While this result implies that both strains are fundamentally different to the parental strain, it does not indicate that the differences to 630 are the same for both strains. The analysis depicted by the Venn diagram (Fig. 7) confirms the results of the PCA, showing that the majority of differentially expressed genes are observed comparing 630Δ*erm* and 630E to 630. From a total of 1337 differentially expressed genes (Table S3 contains all the genes differentially expressed along the growth and also comparisons between the strains), only 139 were common between all 3 strains. A total of 345 were common between 630E and 630Δ*erm*, 60 were common between 630 and 630Δ*erm* and 58 were common between 630 and 630E.

Most of the 345 genes differentially expressed in both, 630Δ*erm* and 630E, were either up or down-regulated in the same way highlighting again how distinct the 2 strains are from the parental strain 630 (Table S3). In TY medium used for the transcriptomic experiments, known as a non-optimal for spore production, a total of 44 sporulation genes were differentially expressed in both 630Δ*erm* and 630E, and all of these were downregulated at 14 and 24 h compared to 6 h. No further differentially expressed sporulation genes appeared in 630Δ*erm*, however, our analysis showed a further 22 sporulation genes, of which 21 were downregulated, in 630E. Among these was the master regulator of sporulation *spo0A*. Nine genes classed as stress-related are differentially expressed in all 3 strains (5 upregulated), with a further 3 in 630 (all upregulated), 7 in 630Δ*erm* (4 upregulated) and 16 in 630E (11 upregulated). Nine genes related to secretion are down regulated in 630E and one gene related to type IV pili is upregulated. In comparison only one secretion gene (putative pilus assembly ATPase) is differentially expressed only in 630Δ*erm* (downregulated) and none in 630. Metabolism is also highly differentially regulated in the 3 strains. 90 genes were uniquely, differentially expressed in 630E, 50 in 630Δ*erm* and 30 in 630. In particular the amino acid metabolism stands out for 630E with the majority of genes being downregulated. (Table S3).

RNAseq data can be used to independently corroborate genome re-sequencing data. Thus, it was apparent that those changes identified by CLC Bio as being present with a frequency of 70% or less, except for CD630_20102, were not real accordingly to the RNAseq analysis (Table S1). This increases the confidence in disregarding changes identified by NGS with a low frequency. In most cases, the SNPs and Indels identified by NGS were confirmed by the RNAseq analysis, with the following exceptions: for SNPs in 630E we found 2 disagreements notably in CD630_33790 and CD630_29430, which both had 100 % frequency and a high coverage (around 200 reads) in the DNA sequence analysis, but only low coverage in the RNAseq experiment. Due to the low coverage of these regions during the RNAseq experiment, which is indicative of low or no expression under the examined conditions, a sequencing error cannot be excluded. For the SNP in CD630_12740 the RNA coverage corroborated the genomic data for 630E, but was in disagreement with the genomic data for 630 and 630Δ*erm*. For three SNPs in 630Δ*erm* similar scenarios were observed. CD630_19070 had very low RNA coverage, CD630_35650 showed ambiguous RNA data with low coverage for 630E. CD630_08260, the *perR* homolog, had convincing DNA data, with frequencies of 98–100 % and coverage of at least a 100 which was corroborated for 630Δ*erm* by RNAseq coverage.

In terms of actual expression data (Table S1), CD630_07610 (the RNA helicase), CD630_14040 (oligopeptide transporter), CD630_20270 (hydrolase) and CD630_29430 (phage replication protein) all showed differential expression in 630E compared to the other 2 strains, with the first 2 showing reduced expression and the latter 2 an increase. CD630_29430, however, also showed an increase in expression in 630 at the later time point. Changes in CD630_26670 in 630Δ*erm* and 630E both seem to lead to severely reduced expression. CD630_12740 (*topA*) only showed differential expression at 24 h in 630Δ*erm* and CD630_12140 (*spo0A*) expression was severely reduced in 630E.

### Discussion

Previously, 2 studies^8,9^ have attempted to use isogenic mutants defective in the production of either toxin A or toxin B to determine the relative importance of these 2 virulence factors in CDI using the hamster infection model. However, despite generating essentially equivalent A+B- insertion mutants in ostensibly the same strain of *C. difficile* (630), contradictory outcomes were obtained in terms of the importance of toxin A. Thus, a *tcdB* mutant created in the one study^8^ producing only TcdA did not cause disease in the hamster, whereas the equivalent ClosTron mutant made in our laboratory (Minton group)^9^ remained virulent. The work undertaken here has provided compelling evidence that the reason for the observed conundrum resides in the use of 2 different erythromycin-sensitive derivatives of strain 630.

Here we have shown that both erythromycin-sensitive derivatives, 630E^14^ and 630Δ*erm*^13^ carry a significant number of SNPs compared to the published sequence. Moreover, it is clear that while the phenotypic properties of 630Δ*erm* and its mutant derivatives closely resemble that of the parent strain 630, strain 630E and its progeny exhibit substantive differences. Thus, whereas latter strains exhibit reduced growth rates, are less proficient in spore formation and are non-motile, 630Δ*erm* strains mirror the behavior of the 630 parental strain with respect to these phenotypes. Furthermore, 630E strains produce reduced amounts of toxin and both struggle to colonize hamsters, and once colonized, animals are less likely to succumb to disease. In short, 630E and its derivatives (i.e., 630E A-B+ and 630E A+B- CT) are less virulent than 630Δ*erm* and its mutant counterparts (i.e., 630Δ*erm* A+B-).

The altered properties of 630E and its derivatives are undoubtedly a consequence of the observed SNPs. However, the substantive number of changes involved makes it difficult to assign any particular SNP to a specific alteration in the observed phenotype, particularly as a combination of mutagenic changes could be responsible. While it is now possible to make precise changes to the genome using allelic exchange methodologies^15^ it is not practically feasible to make all of the sequential rational changes needed to definitively identify the mutation(s) responsible for a particular phenotype. As such, we only corrected 3 specific SNPs that we reasoned may be making a significant contribution. The outcomes of these experiments only emphasized the difficulty of such an undertaking, and served to highlight the dangers involved in making assumptions. Thus, while it seemed reasonable to assume that the DNA inversion within the promoter region of the flagella operon was likely to have caused the observed non-motile phenotype, this surprisingly proved not to be the case. Re-inversion of the 150 bp region failed to restore motility. Clearly other SNPs are at least partly responsible for the observed lack of motility. Singling out any other SNP as the culprit would in the absence of experimental evidence be counterproductive.

Equally negative was the observed outcome of correcting the mutation in CD630_12740 that results in a truncation of the encoded topoisomerase I enzyme. Given this enzyme controls DNA supercoiling, and given that its mutation in certain bacteria is either a lethal event^25-27^ and/or is involved in the regulation of virulence factors,^28-30^ it seemed likely that its presence would result in pleiotropic effects that could have contributed to the observed phenotypic changes. However, its correction, with the exception of a measurable increase in toxin B levels, seemingly had no effects on the behavior of the strain, at least for those properties measured. The reasons are not clear. In other bacteria, mutations of *topA* are only isolated if compensatory mutations arise elsewhere in the genome.^26^ Whether any of the other SNPs present in 630E (eg., the RNA helicase mutation) are negating the effects of the TopA truncation is currently unknown.

To understand the differences observed further, we analyzed the transcriptome of 630, 630Δ*erm* and 630E, comparing expression at 6 h to 14 h and to 24 h (Table S3). The data corroborated the phenotypic analysis, showing vastly different transcriptomes for all 3 strains. While 630E and 630Δ*erm* cluster together in the PCA (Fig. 6), this only highlights how different the 2 strains really are from the progenitor. The analysis clearly shows that the 3 strains are very different from each other and also serves as an explanation as to why the change of a single SNP could not restore any given phenotype. Overall 630E seems the most divergent with many genes differentially expressed involved in metabolism and regulation (Table S3). Additionally 32 genes grouped under the descriptor ‘cell factor’, many of which play a role in energy metabolism, are differentially expressed in 630E, with only 6 of these being upregulated. In contrast out of 19 genes in 630, 11 are upregulated and, out of 10 in 630Δ*erm*, 6 were upregulated. The number of genes downregulated in energy metabolism in 630E might relate to the growth differences seen between the strains.

Interestingly 22 genes involved in sporulation are differentially expressed, 21 of these downregulated, in 630E versus one in 630 and 2 in 630Δ*erm*. This is consistent with the observed delay in sporulation and reduced amount of spores produced by 630E. Secretion also seems most affected in 630E, with 10 genes differentially expressed, compared to none in 630 and one in 630Δ*erm*. A general defect in secretion could affect the secretion of certain virulence or adhesion factors. Furthermore, 33 cell wall genes are differentially expressed in 630E (compared to 14 each in 630Δ*erm* and 630). This may also contribute to the observed colonization deficiencies. Mobile elements are, however, more differentially transcribed in 630 and 630Δ*erm* (15 each) vs. 630E (9). As in many cases different pathways were affected, we propose that this could at least in part explain the different adaptability and virulence of the 2 strains. In both strains many regulators were differentially affected providing a further basis for the observed phenotypic variation between strains.

### Conclusion

Our study has established that the parental strains (630E and 630Δ*erm*) used in the 2 previous studies, that explored the relative roles of toxin A and toxin B in disease,^8,9^ are phenotypically and genetically distinct. Here we also reveal that the 3 strains (630, 630Δ*erm* and 630E) have vastly different transcriptomes, which no doubt lead to the different phenotypes observed. This immense diversity also underlines our finding that by restoring just one SNP, the entire transcriptome cannot be changed. The presence of SNPs in strain 630E significantly affects its transcriptome which in turn has a significant impact on growth, sporulation and finally virulence of this strain in the hamster model of infection under the conditions tested. Data (such as motility, toxicity and virulence) obtained with strain 630Δ*erm* reflects more accurately the behavior of the parent strain 630. As such, it may be concluded that 630 producing toxin A alone will cause disease in the hamster. As a consequence, toxin A should remain a target for the rational development of effective countermeasures against *C. difficile*.

This study has also highlighted a number of issues that need to be borne in mind in the future. At a specific level, if researchers wish to undertake genetic-based studies with strain 630, then the use of strain 630Δ*erm* should be favored over strain 630E. At a more fundamental level, researchers need to effectively curate their strains to prevent the inadvertent isolation of SNPs. Ideally, master seed banks need to be established as frozen glycerol stocks. Moreover, the genome of the stored strain should be re-sequenced as part of the storage process whenever a strain is received from external sources, regardless of whether it has been re-sequenced in the sending laboratory.

## Materials and methods

### Bacterial strains and routine culture conditions

Bacterial strains and plasmids used in this study are listed in Table 2. *E. coli* was cultured aerobically at 37°C with shaking at 200 rpm in LB medium with chloramphenicol supplementation (25 μg/ml) where appropriate. *C. difficile* was cultured in TY (tryptose yeast) medium supplemented with thiamphenicol (15 μg/ml) where appropriate. When needed, *C. difficile* strains were plated on BHIS agar (Brain Heart Infusion agar [Oxoid] supplemented with 5 mg/ml yeast extract [Oxoid] and 0.1% [wt/vol] cysteine [Calbiochem]) supplemented with d-cycloserine (250 μg/ml), cefoxitin (8 μg/ml) [Oxoid] (BHIScc). Fluorocytosine selections were carried out on *C. difficile* minimal medium (CDMM) as described previously.^15^ All *C. difficile* cultures were incubated at 37°C anaerobically in an anaerobic MACS1000 workstation (Don Whitley, Yorkshire, UK).

**Table 2.** Strains and plasmids used in this study.

Name		
Bacterial strains	Description	Source
*E. coli* TOP 10	F– *mcrA* Δ(*mrr-hsdRMS-mcrBC*) Φ80*lac*ZΔM15 Δ*lac*X74 *recA1 araD139* Δ (*ara leu*) 7697 g*alU galK rpsL* (Str^R^) *end*A1 nupG	Invitrogen
*E. coli* CA434	Conjugation donor	Williams et al.^36^
*C. difficile* 630	Wild-type	Brendan Wren
*C. difficile* 630Δ*erm*	Erythromycin sensitive strain of *C. difficile* 630	Hussain et al.^13^
*C. difficile 630*Δ*erm(*Δ*pyrE)*	*C. difficile* 630Δ*erm* containing a deletion in the *pyrE* gene	Ng et al.^19^
*C. difficile* 630E	Erythromycin sensitive strain of *C. difficile* 630	Lyras et al.^8^
*C. difficile* 630Δ*erm* A+B-	*C. difficile* 630Δ*erm tcdB*-1511a::intron *ermB*	Kuehne et al.^9^
*C. difficile* 630E A+B-	*C. difficile* 630E	Lyras et al.^8^
*C. difficile* 630E A+B-CT	*C. difficile* 630E *tcdB*-1511a:: intron *ermB*	This study
*C. difficile* 630E_*topA*	*C. difficile* 630E	This study
*C. difficile* 630E_*flgB*	*C. difficile* 630E	This study
*C. difficile* 630E_CD2667	*C. difficile* 630E	This study
Plasmids		
pMTL007C-E2:*tcdB*-1511a	ClosTron plasmid containing retargeted region to *tcdB* at IS 1511 (antisense oriented) for *C. difficile* 630Δ*erm* or 630E	Kuehne et al.^9^
pMTL-SC7315λ2.3::*topA*	pMTL-SC7315λ2.3 containing 1,000 bp homology arms to change nucleotide 1480649 from T to C in 630E	This study, based on Cartman et al.^15^
pMTL-SC7315λ2.3::*flgB*	pMTL-SC7315λ2.3 containing 1,156 bp homology arms to reverse the inversion upstream of *flgB* in 630E	This study, based on Cartman et al.^15^
pMTL-SC7315λ2.3::CD2667	pMTL-SC7315λ2.3 containing 1,000 bp homology arms to change nucleotide 3079815 from C to A in 630E	This study, based on Cartman et al.^15^

### Mutant nomenclature

For the sake of simplicity, *C. difficile* strains that carried a *tcdA* insertional mutant were referred to as A-, those carrying a *tcdB* mutant as B-, and those strains carrying a mutation in both genes as A-B-. To avoid any ambiguity, if the gene was not inactivated it was referred to as A+ or B+, as appropriate. Thus, a *tcdA* mutant of 630Δ*erm* constructed using ClosTron technology as described,^9,16^ was designated 630Δ*erm* A-B+. The equivalent mutant in 630E constructed through the insertion of a replication-deficient plasmid, according to the method of O'Connor et al.,^14^ was designated 630E A-B+. When ClosTron technology was used in 630E, this was clarified by adding a “CT” suffix, 630E A-B+CT to the strain designation.

### Whole genome sequencing and bioinformatics

Genomic DNA from strains 630E A+B-, 630E A+B-CT and 630Δ*erm* A+B- was prepared by phenol:chloroform extraction. 630E A+B-, 630E A+B-CT, 630Δ*erm*(Δ*pyrE*) were sequenced on Ilumina HiSeq (GATC, Germany) and 630Δ*erm* A+B- on a Roche 454 (Deepseq, Nottingham, UK) and the data compared to the published genome of 630^11,19^ using CLC genomic workbench. All raw sequencing data have been deposited in the sequence read archive (SRA) under the study name PRJNA304508. The accession number is SRP066836. The sequencing data for 630Δ*erm*Δ*pyrE* had been obtained previously^19^ and with no additional changes, other than the *pyrE* deletion, present compared to 630Δ*erm*, were used to analyze the parental strain 630Δ*erm*. We used a frequency of 70% as a cut-off for SNP calling. SNPs, InDels and inversions were confirmed by amplifying a few hundred base pairs up- and downstream of the area of interest (primers are listed in Table S4) and the amplicon was Sanger sequenced (Source BioScience, UK). This confirmation was done on all strains including the parental strains (630, 630Δ*erm*, 630E) and the derivatives (630Δ*erm* A+B-, 630E A+B-, 630E A+B-CT).

### Correction of SNPs and reversal of 150-bp region within the flagellar operon

Using the method described by Cartman et al.^15^ we “corrected” the 2 SNPs and an inversion in 630E to the 630Δ*erm* genotype. A stretch of DNA corresponding to approximately 500 bp either side of the area to be altered was synthesized by Biomatik and cloned into plasmid pMTL-SC7315λ2.3. This vector was transformed by electroporation into *E. coli* CA434 cells^36^ and subsequently conjugated into 630E. Single crossover colonies were identified as those growing faster on plates containing thiamphenicol. Following overnight incubation on CDMM containing 5-fluorocytosine, colonies were incubated on BHIScc plates with and without thiamphenicol. Those strains that had lost the plasmid (both wildtype and double crossover) were unable to grow on thiamphenicol. SNP corrections were confirmed by PCR (Primers see Table S4) and Sanger sequencing (Source BioScience, UK).

### ClosTron mutagenesis

A *tcdB* mutant was generated in the 630E background according to the published method,^16^ using the same plasmid that was used to generate the equivalent mutant in 630Δ*erm*.^9^ This newly created strain was referred to as 630E A+B- CT.

### Southern and western blot

The Southern and Western blot were performed as described in Kuehne et al., 2010.^9^

### *In vivo* testing of mutants

*In vivo* testing was carried out in Syrian Golden hamsters (Charles River, Germany) as previously described.^9^ Briefly, clindamycin was administered orally on day −5 to render the animals susceptible to infection. On day zero, 10,000 spores were administered orally. Animals were assessed for signs of CDI (weight loss, wet tail, lethargy, lack of response to stimulus) 6 times a day for the first 5 days, and once daily for the following 14 d. At this point animals that failed to display signs of CDI were euthanised. Faecal pellets were collected daily from day zero to endpoint, homogenized and plated on *C. difficile* fructose agar (CDFA). *C. difficile* colonies were sub-cultured onto BHIS agar and the genotype was established by PCR (primers in Table S4) followed by Sanger sequencing (results in Table S2). At the experimental endpoint, part of the cecum of each animal was collected. This was also used to plate on CDFA to verify colonisation.

### *In vitro* testing of mutants

**Growth curves**: To assess the effects of SNPs and “corrected” SNPs/inversion on the growth characteristics of all strains, we performed growth curve experiments over 24 h. A 180 μL volume of TY medium was inoculated with 20 μL of an overnight culture in 96-well plates and incubated for 24 h in a GloMax-Multi Microplate Multimode Reader (Promega, USA). Samples were shaken every h and OD_600_ measurements were taken immediately after.

**Motility assays**: 2xYTG (tryptone (1.6%), yeast (1%), NaCl (0.4%), Gelzan (0.24%)[Sigma-Aldrich] and glucose (0.5%)) agar was utilised. 25 mL were poured into each petri dish and let to solidify at room temperature for 15 min. The plates were then dried at 37°C for 30 min. The plates were placed into the anaerobic cabinet 24 h before use. 2 μL volumes of overnight culture were ‘dropped’ onto each plate. Plates were incubated anaerobically for 48 h. Motility was assessed by eye and the plates photographed.

**Sporulation and germination assays**: Sporulation and germination assays were carried out as previously described.^17,18^ Briefly, for the sporulation assay cultures were grown for 5 days, with 2 × 500 µl samples taken at 0, 24, 48, 72, 96 and 120 h. One sample from each time point was heated to 65°C for 30 min while the other sample was kept at room temperature. After this time, samples were serially diluted from 10^0^ to 10^−7^ in PBS. 3 × 20µl of each dilution was spotted onto BHIS plates containing 0.1% taurocholic acid and were incubated for 24 h. The following day, colonies were counted and CFU/mL were calculated. A 630Δ*erm spo0A*::CT mutant strain (containing a ClosTron insertion in the *spo0A* gene^37^), which is unable to form spores, was used as a negative control.

Germination was measured as a function of the ability of a germinated spore to outgrow in the absence of taurocholate. Spore stocks were prepared as previously described by Heeg *et al*., 2012^38^ and stored at −20°C. The optical density of spore suspensions (OD_600_) was adjusted to 1.0 and 450 µl was used per measurement. This equated to approximately 2.5 × 10^7^ spores. Spore suspensions were heat treated at 60°C for 25 min to kill any remaining vegetative cells and then centrifuged and resuspended in BHIS with the germinant taurocholic acid (0.1%) in a total volume of 20 ml. Samples were taken at 5, 10, 20, 30, 60, 90, 120, 180 and 240 min, briefly centrifuged, washed and resuspended in phosphate-buffered saline (PBS), the samples were then diluted and plated on plain BHIS agar. Plates were incubated for 24 h before the CFUs were enumerated. Colonies that grew on these plates were considered to be germinated vegetative bacteria.

**Toxin A/B ELISA**: ELISA assays were performed using the *C. DIFFICILE TOX A/B II*™ kit from TechLab and the TGC-E002-1-separate detection of *C. difficile* toxins A and B kit from TGCbiomics according to the manufacturers' instructions. Cultures of *C. difficile* were grown in TY medium without glucose for 72 h, at which time 1 ml samples were taken, centrifuged and the supernatant filter-sterilised and used for the ELISAs. A 1:2 dilution was used for the toxin B ELISA kit from TGCbiomics. To quantify toxins a standard curve with pure toxin (the native antigen company) was established for the TechLab ELISA and also Toxin A ELISA from TGCbiomics.

**RNAseq**: Total RNA was extracted from 2 independent biological replicates of 630, 630Δ*erm* and 630E strains at 3 time points (18 samples). Bacteria were grown in TY broth medium after 6, 14 and 24 h as previously described.^35^ The mRNA was treated with MicrobExpress kit (Ambion). For oriented RNA-seq library construction, the Truseq stranded RNA seq Illumina kit was used according to manufacturer's instructions before sequencing using the Illumina HiSeq 2500 machine. Sequencing reads were mapped using Bowtie.^39^ to the reannotated 630 reference genome^40^ complemented with the known ncRNA.^41^ Statistical analyses were performed on each strand coverage count with DESeq2^42^ using the 6 h value as a reference for reporting the expression data of 14 and 24 h. A gene was considered differentially expressed when the fold change was > 2 and the *P value* was < 0.05.

The RNA-seq data discussed in this publication have been deposited in NCBI's Gene Expression Omnibus database under the accession no. GSE72006

RNA-seq coverage visualization is available through the COV2HTML software:^43^

**Table:** 

630	14H - 6H	http://mmonot.eu/COV2HTML/vizualization.php?str_id=-20
	24H - 6H	http://mmonot.eu/COV2HTML/vizualization.php?str_id=-22
630Δ*erm*	14H - 6H	http://mmonot.eu/COV2HTML/vizualization.php?str_id=-24
	24H - 6H	http://mmonot.eu/COV2HTML/vizualization.php?str_id=-26
630E	14H - 6H	http://mmonot.eu/COV2HTML/vizualization.php?str_id=-28
	24H - 6H	http://mmonot.eu/COV2HTML/vizualization.php?str_id=-30

### Graphs and statistical analyses

All graphs were generated and statistical analyses were performed using GraphPad PRISM 6.02. Statistical analysis comprised either 2-way ANOVA for multiple comparisons or unpaired t test for pairwise comparisons. All experiments were carried out in triplicate unless stated otherwise.

### Ethics statement

This work was reviewed and approved locally by the Animal Welfare and Ethical Review Body (formerly the Ethical Review Committee) at the University of Nottingham and performed under a project license (PPL 40/3590) granted under the Animal (Scientific Procedures) Act, 1986, by the UK Home Office. The work was performed in accordance with the NC3R^s^ ARRIVE guidelines.^44^

## Supplementary Material

KVIR_S_1237333.zip
