# Supplementary material for: What's a SNP between friends: The influence of single nucleotide polymorphisms on virulence and phenotypes of Clostridium difficile strain 630 and derivatives
Source: Virulence. 2016 Sep 21;8(6):767–81. doi: 10.1080/21505594.2016.1237333 (PMC5626343; doi:10.1080/21505594.2016.1237333)
Supplement: KVIR_S_1237333.zip [file kvir-08-06-1237333-s001.zip › KVIR_S_1237333.docx]

**Supporting Information**

Figure S1. Southern and Western blot of 630E A+B- CT. A. Southern blot showing the single ClosTron insertion to create 630E A+B- CT (mutant 1, 2 and 3). The ClosTron plasmid pMTL007C-E2:*tcdB*-1511a was used as a positive control and the parental strain 630E as a negative control. The probe used binds to the intron insertion. B. Western blot using an anti-TcdA antibody to detect production of toxin A in the supernatant of 630, 630Δ*erm*, 630Δ*erm* A+B-, 630E, 630E A+B- and 630E A+B- CT after 96 h.

**Table S1. Genomic and transcriptomic differences between 630, 630Δ*erm* and 630E.**

SNPs, insertions (INS) and inversion (INV) are shown in this table as well as the corresponding RNAseq coverage. Changes between 630, 630Δ*erm* and 630E are represented compared to the published genome of 630, the new 630 genome by Riedel *et al*. [^22^](#_ENREF_22) and also the newly published genome of 630Δ*erm* [^23^](#_ENREF_23).

See attached excel file: S1_Table

**Table S2.** **Colonisation of hamsters with *C. difficile* strains.**

Colonisation of hamsters with 630E, 630E A+B- and 630E A+B- CT was confirmed by plating faecal and caecal samples on CDFA and by extraction genomic DNA and PCR of the toxin A and B genes.

| **Strain given** | **Colonisation** | **630 PCR** | ***tcdA*** | ***tcdB*** | **Succumbed to CDI** |
| --- | --- | --- | --- | --- | --- |
| 630E |  | Caecum | + | + |  |
| 630E | Colonised to Day 18 | Faecal | + | + |  |
| 630E | Colonised to Day 15 | Faecal | + | + |  |
| 630E |  | Caecum | + | + | CDI |
| 630E |  | Caecum | + | + | CDI |
| 630EA+B- |  | Caecum | + | - | CDI |
| 630EA+B- | Not colonised | Faecal | - | - |  |
| 630EA+B- | Not colonised | Faecal | - | - |  |
| 630EA+B- | Not colonised | Faecal | - | - |  |
| 630EA+B- | Not colonised | Faecal | - | - |  |
| 630EA+B- |  | Caecum | + | - |  |
| 630EA+B- |  | Caecum | + | - | CDI |
| 630EA+B- |  | Caecum | + | - |  |
| 630EA+B- | Colonised to Day 3 | Faecal | + | - |  |
| 630EA+B- |  | Caecum | + | - |  |
| 630EA+B- |  | Caecum | + | - |  |
| 630E A+B- CT |  | Caecum | + | CT | CDI |
| 630E A+B- CT |  | Caecum | + | CT | CDI |
| 630E A+B- CT | Colonised to Day 15 | Faecal | + | CT |  |
| 630E A+B- CT | Colonised to Day 18 | Faecal | + | CT |  |
| 630E A+B- CT |  | Caecum | + | CT |  |
| 630E A+B- CT |  | Caecum | + | CT |  |

**Table S3. Transcriptomic differences between 630, 630Δ*erm* and 630E.**

Differentially expressed (DE) genes, comparing RNAseq data from 6 h to 14 h and 24 h in 630, 630Δ*erm* and 630E. The genes are classed into cell factor, cell growth, cell wall, fermentation, membrane transport, amino acid metabolism, carbon metabolism, cofactor metabolism, lipid metabolism, nucleic acid metabolism, mobile elements, motility, operons, regulation, anaerobic respiration, secretion, sporulation, stress, translation, unknown and virulence factors. Downregulated genes are coloured in green and upregulated genes in red.

See attached excel file: S3_Table

**Table S4. Primers used in this study.**

| **Primer name** | **Sequence (5’-3’)** | **Explanation or SNP target (where applicable)** |
| --- | --- | --- |
| topAMC1 | GATGCACAACAGGCAAGAAGAGTGC | To screen for chromosomal change |
| topAMC2b | CCCGATTGTAAAACAACTAGACCAATTATG | To screen for chromosomal change |
| flgBMC1b | ctatcaaatacagatggaagttgtggtg | To screen for chromosomal change |
| flgBMC2c | CGAGCATATGATTCTAACGTAGATACATTGAATG | To screen for chromosomal change |
| CD2667MC1 | gtcaccttatgagtgaagtttgtaataaatgtgg | To screen for chromosomal change |
| CD2667MC2b | gcaagagctgctgctggaagac | To screen for chromosomal change |
| JRP3441 ^8^ | GTTACCAGGAATACAACCAGAC | To test for interrupted *tcdB* |
| JRP2839 ^8^ | CGGCCAGCCTCGCAGAGCAG | To test for interrupted *tcdB* |
| JRP3442 ^8^ | GCACTTGCTTGATCAAAGCTCC | *toxA* specific PCR, positive for all strains (630E, 630E A+B-, 630E A+B-CT) |
| JRP2342 ^8^ | CCGGAATTCGCTCTATTGGACTAGACCGTTG | *toxA* specific PCR, positive for all strains (630E, 630E A+B-, 630E A+B-CT) |
| Cdi-tcdB-F1 ^9^ | TGATAGTATAATGGCTGAAGCTAATGCAGATAATGG | To test for *tcdB* ClosTron insertion |
| Cdi-tcdB-R1 ^9^ | CTTGCATCGTCAAATGACC ATAAGCTAGCC | To test for *tcdB* ClosTron insertion |
| 268934_F | tgtcaagtgaattagaaagaaacca | 268934 |
| 268934_R | aagtgagccgtgttttgaaaa | 268934 |
| 309208_F | gccagttgccaaaaagagtc | 309208 |
| 309208_R | ggcatagcatcatttagtgtttc | 309208 |
| MCSNP11 | cgggaaaaacagctgcttttagtatccc | 933139 |
| MCSNP12 | cctcttgcttgtaaatctcctaccaattc | 933139 |
| 10000995_F | gatgaagaagttgtttggcaat | 1000995 |
| 10000995_R | Cctacttggctacaccttttaca | 1000995 |
| 1391850_F | Tctgtcatttggaaaggatgaa | 1391850 |
| 1391850_R | Tctgtacttgcttttgatatacttgga | 1391850 |
| Spo0A_F1 | GGCATAGCTAAGGATGGAATTG | 1413057 |
| Spo0A_R1 | GGAGTAGAGGAAAAGTTGACACAA | 1413057 |
| 1480649_F | gcttcaacaagaaggagcaaa | 1480649 |
| 1480649_R | tgctggtggttgtgtaaaatg | 1480649 |
| 1607453_F | Ttgaaggtgtaaactcagttgtagg | 1607453 |
| 1607453_R | Tccaaataaaagtctatgaaaatgaa | 1607453 |
| 1626977_F | tggtggtagcaaaaacgaaa | 1626977 |
| 1626977_R | tgccattgaatttgttgcag | 1626977 |
| 2044514_F | catactaaatgaggggtaaaataaaga | 2044514 |
| 2044514_R | tttttctgccttttctctttgtg | 2044514 |
| MCSNP1 | gttctgtaatacctttttctttagctattttaattgc | 2203033 |
| MCSNP2 | gttaccgatattataggcaaaactgccc | 2203033 |
| 2209236_F | ccattagtgagtgatgatttacttcc | 2209236 |
| 2209236_R | gcaagttttgctatttctctttctt | 2209236 |
| 2320410_F | Gtcactggtaggaattaatctaacg | 2320410 |
| 2320410_R | Tgctttcacaaatgctttcg | 2320410 |
| MCSNP13 | gaaagatgaatttctatccatcttcatcaaatgtgg | 2339506 |
| MCSNP14 | ggggctgttgaccttggaccc | 2339506 |
| MCSNP3 | cctttgatgtctagttaatttcttcacttattttaagc | 2937176 |
| MCSNP4 | ggaaaaccagcaaaagcttgtattatgattccc | 2937176 |
| 3005866_F | aatataattcccaaccttccaaa | 3005866 |
| 3005866_R | tttgtttgaagattagtggtgattg | 3005866 |
| MCSNP5 | GTATATTTTTTCTCTAGCTTTATCTCCATCAGGG | 3034953 |
| MCSNP6 | ggaaaggataaaccaggtatagtggc | 3034953 |
| 3079815_F | cccgcttttacttcatctcc | 3079815 |
| 3079815_R | gcatcagagattttgattgcttt | 3079815 |
| MCSNP7 | cctgctacaaattttttcttttctggc | 3080703 |
| MCSNP8 | ctgcttatctttataaaaagttttataaaattgaattacctc | 3080703 |
| MCSNP15 | cttcatattgagtgaaagtctgattgaagttagc | 3422569 |
| MCSNP16 | ctcaaccgtgtgccgttttcccg | 3422569 |
| 3526888_F | ctctttcctgcattcccaag | 3526888 |
| 3526888_R | Ttgttgagcagatataaaatccca | 3526888 |
| MCSNP17 | caatctattcaaagataaactatagtacttcttctac | 3528736 |
| MCSNP18 | cctactcctttaggtgtgagatgg | 3528736 |
| 3591103_F | ggcactagctgctcctaataaa | 3591103 |
| 3591103_R | ccatatacccctatccctcctt | 3591103 |
| 3686535_F | tcttccaagctcttacctgtttg | 3686535 |
| 3686535_R | Gctctgtccagttaattg | 3686535 |
| 3797112_F | tgctcctgtaaatgcacctg | 3797112 |
| 3797112_R | Ctgtaaaatacaagtcactcattccaa | 3797112 |
| MCSNP19 | ccgttccagactgttcaatgctcc | 3951559 |
| MCSNP20 | cctaagtagtagttactggcaacagcac | 3951559 |
| MCSNP9 | gcacccttaataacttgaccagttaaaaagg | 4007463 |
| MCSNP10 | cgcccgaagccgattatctaacc | 4007463 |
| 4166495_F | gcatcaagtaagtatttatgctcttca | 4166495 |
| 4166495_R | Tgaacttggataattacaagccatt | 4166495 |
